# Research and Application of Marine Microbial Enzymes: Status and Prospects

**DOI:** 10.3390/md8061920

**Published:** 2010-06-23

**Authors:** Chen Zhang, Se-Kwon Kim

**Affiliations:** 1 Department of Chemistry, Pukyong National University, Busan, 608-737, Korea; 2 Key laboratory of Molecular Enzymology and Enzyme Engineering of Ministry Education, Jilin University, Changchun, 130023, China; E-Mail: ahxczc@yahoo.cn; 3 Marine Bioprocess Research Center, Pukyong National University, Busan, 608-737, Korea

**Keywords:** marine microbial enzymes, protease, lipase, polysaccharide-degrading enzyme, extremozymes

## Abstract

Over billions of years, the ocean has been regarded as the origin of life on Earth. The ocean includes the largest range of habitats, hosting the most life-forms. Competition amongst microorganisms for space and nutrients in the marine environment is a powerful selective force, which has led to evolution. The evolution prompted the marine microorganisms to generate multifarious enzyme systems to adapt to the complicated marine environments. Therefore, marine microbial enzymes can offer novel biocatalysts with extraordinary properties. This review deals with the research and development work investigating the occurrence and bioprocessing of marine microbial enzymes.

Enzymes have been isolated and purified from microorganisms, animals and plants; among them microorganisms represent the most common source of enzymes because of their broad biochemical diversity, feasibility of mass culture and ease of genetic manipulation [1–3]. The world’s oceans cover more than 70% of our planet’s surface; countless marine microorganisms contain biochemical secrets which can provide new insights and understanding of enzymes. Marine microorganisms have been attracting more and more attention as a resource for new enzymes, because the microbial enzymes are relatively more stable and active than the corresponding enzymes derived from plants or animals[4, 5]. With the recent advent of biotechnology, there has been a growing interest and demand for enzymes with novel properties. When compared with the terrestrial environment, the marine environment gives marine microorganisms, unique genetic structures and life habitats [6]. The marine environment ranges from nutrient-rich regions to nutritionally sparse locations where only a few organisms can survive. The complexity of the marine environment involving high salinity, high pressure, low temperature and special lighting conditions, may contribute to the significant differences between the enzymes generated by marine microorganisms and homologous enzymes from terrestrial microorganisms, leading to the boosted marine microbial enzyme technology in recent years and the resulting valuable products. These enzymes are used as pharmaceuticals, food additives, and fine chemicals [7–10]. In recent years, researchers have isolated a variety of enzymes with special activities from marine bacteria, actinomycetes, fungi and other marine microorganisms, and some products have already been used in industrial applications. In particular, some marine microbial enzymes have yielded a considerable number of drug candidates. Marine microorganisms, whose immense genetic and biochemical diversity is still in its infant stage, are of considerable current interest as a new promising source of enzymes with unsuspected application potentials [11,12].

## 1. Protease

Total protease sales represent more than 60% of all industrial enzyme sales in the world. In modern society, the proteases are widely used. Proteases are used in the detergent industry, leather industry, and also for pharmaceutical applications, such as digestive drugs and anti-inflammatory drugs [13–18]. In 1960, Dane first isolated alkaline protease from *Bacillus licheniformis*. So far, it is still found that microorganisms are the most suitable resources for protease production. In 1972, Nobou Kato isolated a new type of alkaline protease from marine *Psychrobacter*, and since then quite a few proteases have been continually obtained from marine microorganisms. An alkaline protease, previously isolated from a symbiotic bacterium found in the Gland of Deshayes of a marine shipworm, was evaluated as a cleansing additive [19]. Qiu *et al.* selected 30 kinds of marine bacteria from the sea water, mud, fish and other samples; after UV mutagenesis they isolated the N1–35 strain, this strain produced protease that had significant advantages compared with the terrestrial ones [20]. A yeast strain (*Aureobasidium pullulans*) with a high yield of alkaline protease was isolated from sea saltern of the China Yellow Sea by Chi *et al.* in 2007, and the maximum production of enzyme was 623.1 U/mg protein (7.2 U/mL) [21]. In 2009, *Bacillus mojavensis* A21 producing alkaline proteases was isolated from seawater by Haddar *et al*., and they purified two detergent-stable alkaline serine-proteases (BM1 and BM2) from this strain. Both proteases showed high stability towards non-ionic surfactants. In addition, both of them showed excellent stability and compatibility with a wide range of commercial liquid and solid detergents[22].

## 2. Lipase

Lipases are ubiquitous enzymes that catalyze the breakdown of fats and oils with subsequent release of free fatty acids, diacylglycerols, monoglycerols and glycerol. Besides this, lipases are also efficient in various reactions such as esterification, transesterification and aminolysis [23]. Lipases have received increased attention recently, as evidenced by the increasing amount of information about lipases in the current literature. Also, many microbial lipases are available as commercial products, the majority of which are used in detergents, paper production, cosmetic production, food flavoring, organic synthesis and some other industrial applications [24–26]. The enzyme detergent market share has currently reached 90% in Europe, and in Japan around 80%. Lipases are valuable biocatalysts, because they act under mild conditions and are highly stable in organic solvents, show broad substrate specificity[27–29].

With the exploitation of marine resources, the pelagic fishes have become the primary target of fishery: these species are resourceful and potential. However, the fat content is high in these species; people have to face the certain difficulties for fish preservation, processing, marketing. Currently, the traditional methods for fish degreasing include expression method, extraction method and alkaline processing, while compared with these traditional methods, using lipases has incomparable advantages. Therefore, applications of lipases in the fish processing field are causing growing concern[30,31].

Microbial lipase was first found from *Penicillium Oxalicum* and *Aspergillus flavus* in 1935 by Kirsh [32]. Feller *et al.* (1990) screened four cold-adapted lipases secreted by *Moraxella*. These *Moraxella* were obtained from the Antarctic seawater with the optimum growth temperature of 25 ºC, and the maximum secretion of lipases was supposed to occur at lower temperature conditions, the lowest secretion temperature can reach 3 ºC [33]. Wang *et al.* screened out nine lipase producing strains from a total of 427 yeast strains. They belonged to *Candida intermedia* YA01a*, Pichia guilliermondii* N12c*, Candida parapsilosis* 3eA2*, Lodderomyces elongisporus* YF12c*, Candida quercitrusa* JHSb, *Candia rugosa* wl8, *Yarrowia lipolytica* N9a*, Rhodotorula mucilaginosa* L10-2 and *Aureobasidium pullulans* HN2.3. Some lipases could actively hydrolyze different oils, indicating that they may have potential applications in industry [34]. In 2009, a novel extracellular phospholipase C was purified from a marine *streptomycete*, which was selected from approximately 400 marine bacteria by Mo *et al.* Its enzyme activity was optimal at pH 8.0 at 45 ºC, and it hydrolyzed only phosphatidylcholine[35].

## 3. Polysaccharide-Degrading Enzymes

### 3.1. Chitinase and chitosanase

Chitin is widely distributed in nature as a biopolymer with non-toxic properties. After cellulose, it is the most common polysaccharide found in nature, and is the major structural component of most fungi cell walls and also quite abundant in the crust of insects and crustaceans. In nature, annual generation of chitin is about 1.0 × 10^10^ t [36,37]. Chitin and chitosan have a similar chemical structure. Chitin is made up of a linear chain of acethylglucosamine groups, and chitosan is obtained by removing enough acetylglucosamine residues. After hydrolysis, chitin and chitosan could enhance immune function, promote digestive function and eliminate toxins from the body, even inhibit tumor cell growth as well as being involved in other important physiological functions [38–41]. Therefore, hydrolysis of chitin and chitosan recently became a hot topic.

As marine zooplankton are regularly supposed to shed, there is a large amount of abandoned chitin, which could be a rich source of carbon and energy for growth and reproduction of chitin-degrading microorganisms. The total production of chitin in the whole marine biocycle is at least 2.3 million metric tons per year [42]. Until now, researchers have found a wide range of microorganisms that can produce chitinase or chitosanase, including *Aspergillus, Penicillium, Rhizopus, Myxobacter, Sporocytophaga, Bacillus, Enterobacter, Klebsiella, Pseudomonas, Serratia, Chromobacterium, Clostridium, Flavobacterium, Arthrobacter* and *Streptomyces* [43–45]. Osawa *et al.* found chitinase from six species of marine bacteria, *Vibrio fluvialis, Vibrio parahaemolyticus, Vibrio mimicus, Vibrio alginolyticus, Listonella anguillarum* and *Aeromonas hydrophila* [46]. In addition, a variety of chitinase genes were already cloned from marine bacteria and fungi. Suolow and Jone inserted two chitinase genes (ChiA, ChiB) into *E. coli*, and subsequently these genes ware transferred into *Pseudomonas*; finally they acquired four high-yielding chitinase strains [47]. Meanwhile, Roberts and Cabib put the chitinase gene into tobacco plant cells, and were able to develop a new tobacco plant with a strong disease resistance to the pathogen *Alternaria Longipes*[48].

### 3.2. Alginate lyases

The brown alga is one of the largest marine biomass resources. Alginate has a wide range of applications; further, the degraded low-molecular fragment shows more potential. Alginate lyases, characterized as either mannuronate or guluronate lyases, are a complex copolymer of α-l-guluronate and its C5 epimer β-d-mannuronate. They have been isolated from a wide range of organisms, including algae, marine invertebrates, and marine and terrestrial microorganisms. In recent years, the marine microbial alginate lyases have been greatly developed. Discovering and characterization of alginate lyases will enhance and expand the use of these enzymes to engineer novel alginate polymers for applications in various industrial, agricultural, and medical fields[49–53].

### 3.3. Agarases

Agar is a highly heterogeneous polysaccharide. Neutral agarose is an alternating polymer of d-galactose and 3,6-anhydro-l-galactose linked by alternating β4 → 1 and α1 → 3 bonds. Agar oligosaccharides have a wide range of applications in the food industry; it can be used for beverages, bread, and some low-calorie food production. Japanese use agar-oligosaccharide as a moisturizing cosmetic additive, and it also has good hair conditioning effects [54–57].

Nowadays, the acid degradation of agar is replacing enzymatic degradation with the advantages of easy control and mild reaction. Agarase is an enzyme found in agarolytic microorganisms [58]. Agar-degrading microorganisms can be divided into two groups: bacteria soften the agar; the other violently liquifies the agar. In 1902, Gran isolated agar-degrading *Pseudomonas galatica* from seawater. Until now, researchers have found the presence of agarase from species within the genus *Cytophaga, Bacillus, Vibrio, Alteromonas, Pseudoalteromonas, Streptomyces* [59–61]. Susgano *et al.* reported a marine bacterium *Vibrio* sp. (JT0107), which can hydrolyze the α-l,3 glycosidic bond of agar by α-Neoagaro-oligosaccharides [62]. Several agarase genes have been cloned and sequenced. In 1987, Mervyn found the *Streptomyces* agarase gene (dagA) [63]. Soon after, Rosert performed gene sequence analysis of the *Pseudomonas* agarase gene (agrA) [64]. In 1993, the *Vibrio* agarase gene agaA was cloned and sequenced by Yasushi. This agarase hydrolyzes not only agarose but also neoagarotetraose to yield neoagarobiose. This is a unique characteristic for a β-agarase [65]. In 1994, from the same species of bacteria, a new β-agarase gene (agaB) was sequenced by the same group [66]. A new β-agarase was purified from an agarolytic bacterium, *Bacillus* sp. MK03 in 2003 by Suzuki *et al.*; this enzyme could hydrolyze neoagarohexaose to produce neoagarotetraose and neoagarobiose [67]. Researches also demonstrated that glucose can inhibit extracellular agarase secretion without transcription inhibition [68].

### 3.4. Carrageenases

Carrageenan and carrageenin are a family of linear sulfated polysaccharides, which are extracted from red seaweeds. 80% of the carrageenan is used in food and food-related industries, and it can be used as a coagulant, adhesive, stabilizer and emulsifier. In addition, it has also been widely applied in the pharmaceutical and cosmetics industries. The oligosaccharides obtained from carrageenan degradation show a variety of specific physiological activities, such as anti-viral, anti-tumor, anti-coagulation, *etc.* [69,70]. As early as 1943, Mori extracted carrageenase from marine mollusc. Right now, *Pseudomonas, Cytophaga, Alteromonas atlantica, Alteromonas carrageenovora,* and some unidentified strains have been found to possess the carrageenan-degrading enzymes. Sarwar *et al.*, using carrageenan containing medium, cultured *cytophaga* lk-C783, and obtained extracellular κ-carrageenase with a molecular weight of 10 kD [69]. In 2004, Mou *et al.* isolated an extracellular κ-carrageenase with a molecular weight of 30 kD from marine *Cytophaga* MCA-2 [71]. A distinct λ-Carrageenan-degrading *Pseudoalteromonas* bacterium (CL19)was isolated from a deep -sea sediment sample in 2006 by Yukari and Yuji; the molecular mass of this purified enzyme was approximately 100 kD[72].

### 3.5. Cellulose and hemicellulose hydrolase

Cellulose is an organic compound with the formula (C_6_H_10_O_5_) _n_; a polysaccharide consisting of a linear chain of several hundred to over ten thousand β (1→4)-linked D-glucose units. Hemicellulose is a polysaccharide related to cellulose, and in contrast to cellulose, it can be derived from several sugars including glucose, xylose, mannose, galactose, rhamnose, and arabinose. Hemicellulose consists of shorter chains of around 200 sugar units [73,74]. Cellulose is the most available saccharide in nature and is about 50% of all plant matter, and hemicellulose is ~20–30%, while the remainder is mainly lignin[74,75].

Cellulolysis is the process of breaking down cellulose through a hydrolysis reaction into smaller polysaccharides called cellodextrin. Because cellulose molecules strongly bind to each other, cellulolysis is relatively difficult when compared to the breakdown of other polysaccharides [76]. Until now, it was found that bacteria can produce cellulase, including: *Cytophaga, Cellulomonas, Vibrio,* and *Clostridium, Nocardia, Streptomyces*, and for certain fungi it was found that *Trichoderma, Aspergillus, Fusarium, Chaetomium, Phoma, Sporotrichum, Penicillium, etc*. are also able to produce cellulase. Hemicellulase, generally refers to the hydrolase, which can hydrolyze polysaccharides, for example, xylanase, galactanase, arabanase, among which xylanase has particular economic value[77–79].

Cellulase can be used for bio-textile auxiliaries, cotton and linen products processing and bio-fertilizer processing. With the rapid development of the seaweed industry, a mass of waste released into the environment led to very serious pollution problems. Cellulases degrade seaweed processing waste to low molecular fragments, which can be easily absorbed by plants as bio-fertilizer.

Xylanases are hydrolases depolymerizing the plant cell component xylan, the second most abundant polysaccharide. Xylanases could be produced by fungi, bacteria, yeast, marine algae, *etc*., but the principal commercial source is filamentous fungi. Xylanase could be used on semi-cellulose to produce products with high economic value, such as xylitol. In the paper and pulp industry, using xylanase can improve the lignin dissolution rate and reduce the usage of Cl_2_ and ClO_2_, thereby reducing pollution and improving pulp properties. Xylanase can also degrade some polysaccharides in juice or beer, thus it could contribute to beverage clarification [74,75,77,79]. Indian researchers obtained several fungal isolates from marine habitat showed alkaline xylanase activity. The crude enzyme from NIOCC isolate #3 (*Aspergillus niger*) with high xylanase activity, cellulase-free and unique properties containing 580 UL^−1^ of xylanase [80]. Yin *et al.* purified xylanase, which had an optimal pH and temperature at 5.0 and 50 ºC from bacterium *Bacillus* sp. YJ6 [81]. A novel cold-adaptive xylanolytic *Penicillium* strain FS010 was isolated from China Yellow sea sediments by Hou *et al.*, this fungus grew well from 4 ºC to 20 ºC, but a lower (0 ºC) or higher (37 ºC) temperature limited its growth. Compared with the mesophilic *Penicillium chrysogenum*, the cold-active xylanase showed high hydrolytic activities at low temperature (2–15 ºC) and high sensitivity to high temperature (>50 ºC)[82].

### 3.6. Other polysaccharide hydrolases

Amylases were found in bread making, and they can breakdown complex sugars such as starch into simple sugars such as glucose, maltose and dextrin. They can be classified into α-amylase, β-amylase and γ-amylase. Unlike the other forms of amylase, γ-amylase is most efficient in acidic environments. To date, researchers have found some terrestrial microorganisms which can produce extracellular amylase, such as *Arxula adeninivorans, Lipomyces, Saccharomycopsis, Schwanniomyces, Candida japonica and Filobasidium capsuligenum* [83]. With the development of marine science and technology, researchers reported more and more microorganisms from marine habitats capable of producing amylase. The marine yeast strain *Aureobasidium pullulans* N13d, producing an extracellular amylase, was isolated from the deep sea sediments of Pacific Ocean [84]. Chakraborty *et al.* reported a novel α-amylase from marine *Streptomyces* sp. D1 by using media containing 2% sucrose, 0.35% peptone and 0.15% of malt extract [85]. Mohapatra *et al.* isolated a novel amylase from the *Mucor* sp. associated with the marine sponge *Spirastrella* sp., this enzyme has an optimum pH of 5.0 and an optimum temperature of 60 ºC [86].

Fucoidan is a complex sulfated polysaccharide, constituted of fucose, galactose, xylose, mannose, arabinose, and uronic acid. The fucoidan oligosaccharide, with molecular weight <1000 Da, can be used as a human epidermal keratinocytes activator. Furukawa purified fucoidanase, generated from marine *Vibrio*, and obtained three kinds enzymes, which can hydrolyze substrates to small molecule-oligosaccharides[87]. Yaphe and Morgan reported that the marine bacteria *Pseudomonas atlantica* and *Pseudoalteromonas carrageenovora* were cultured in medium for three days by using fucoidan as the sole carbon source, with the substrate utilization as 31.5% and 29.9%, respectively[88].

Japanese researcher isolated *Bacillus circulans* from Tokyo Bay sea mud, which do not grow in conventional culture medium, but when the medium was properly diluted, it could grow and produce a new glucanase. This glucanase can act on the α-1,3 bond and α-1,6 bond in glucan. From the marine *Bacillus,* a new glucanase has been isolated, which showed optimum activity at 37 ºC, and this property is suitable for oraland other health care [89].

Araki *et al.* isolated 117 marine bacteria, belonging to *Pseudomonas, Alcaligenes, Klebsiella, Enterobacter, Vibrio, Aeromonas, Moraxella, Bacillus, etc.*, which can generate β-mannanase [90].

## 4. Extremozymes

The marine environment is extremely complex, including low-temperature, high-temperature, high hydrostatic pressure, strong acid, strong alkali, and very poor nutritional conditions. An extremophilic microorganism is a microorganism that thrives in, and may even require, physically or geochemically extreme conditions that are detrimental to the majority of life on Earth. Extremophilic microorganisms are adapted to survive in ecological niches, they must adaptively change their physiological structure and metabolism, in order to adapt to the extreme environmental conditions. (Table 1) Therefore, extremophilic microorganisms screened from these environments, may have some specific physiological principles, which can produce unique biocatalysts that function under extreme conditions comparable to those prevailing in various industrial enzymes. Because of this reason, in recent years, research programs investigating extremophilic microorganisms became a new area of interest in microbial research and are very popular[91–94].

*Algoriphagus, psychrotrophs* and other low-temperature microorganisms have obvious advantages in the ecology. The low-temperature microorganisms cannot readily be involved in contamination. Their culture condition is simple, and the enzymes from these microorganisms have advantages of high activity and high catalytic efficiency. Hence, with the assistance of low-temperature microbial enzymes, it can greatly shorten the processing time and save expensive heating/cooling systems, thus save considerable energy. Cold-adapted enzymes from marine microorganisms, especially, the lipases and proteases, have considerable potential, particularly in the cleaning industry. Many studies have shown that about 77% of Antarctic marine bacteria are resistant to cold environments and 23% are addicted to a cold environment. The unique geography and climate characteristics in Antarctica forms a dry, bitterly cold, strong radiation environment, in which microorganisms have to survive with corresponding unique molecular mechanism, physiological and biochemical characteristics. For these reasons, the Antarctic marine bacteria are thought to produce new bioactive substances with significant potential [95]. In 1994, Feller screened α-amylase-generating bacteria *Alteromonas haloplanktis* from the Antarctic, which can grow well at 4 ºC, however cell proliferation and enzyme secretion is suppressed at 18 ºC; mean while, at 0 ~30 ºC the activity of this α-amylase is seven-times higher than α-amylases from homeothermic animals [96]. Kolenc transferred TOL plasmid pWWO of mesophilie *Psychrotrophs putida* PaW1 to the psychrotroph *Psychrotrophs putida* Q5. From expression of the genes, it was shown that the transconjugant had the capacity to degrade and utilize toluate (1,000 mg/liter) as a sole source of carbon at temperatures as low as 0 °C [97]. Transferring the useful gene from mesophilic microorganisms to psychrophilic ones was established to promote low-temperature microbial biological features, which may have enormous potential in removing pollution in cold environments.

Near the deep sea volcanoes, some microorganisms can survive in extreme conditions, even over temperatures of 100 ºC. Therefore, these microorganisms are supposed to have unique enzyme systems, which can work in these high temperature conditions. For example, the nucleic acid enzymes, such as DNA polymerase, ligase and restriction endonuclease, have a significant applicational value in molecular biology research. Iundberg’s group purified a thermostable DNA polymerase from thermophilic archaea (*Pyrococcus furiosus*), which has polymerizing and proofreading double functions, and has high activity even at 100 ºC. Hence, this polymerase can be applied in high fidelity PCR experiments [98,99]. In 2008 a novel thermostable non-specific nuclease from thermophilic bacteriophage GBSV1 was isolated by Song’s group, this non-specific nuclease can degrade various nucleic acids, including RNA, single-stranded DNA and double-stranded DNA[100].

Studies have shown hydrostatic pressure can obviously promote enzyme thermal stability. Generally, enzymes have a good three-dimensional specificity under hydrostatic pressure, but when pressure exceeds a certain range, the enzyme’s weak bond can be easily destroyed, thereby leading to the disintegration of the enzyme conformation. The deep-sea is regarded as an extreme environment with conditions of high hydrostatic pressure, Enzymes from deep-sea microorganisms are thought to have characteristic pressure-adaptation mechanisms in structure and function, and they can be utilized in high hydrostatic pressure environments without disintegration. Microorganisms obtained from deep-sea environments appear to be an important source of modern enzyme industries. In 1979, the first barophilic bacteria was isolated from a deep-sea sample and has been found to grow optimally at about 500 bars and 2–4 ºC. Japanese scientists isolated multiple strains of bacteria addicted to pressure from the marine environment, and found that the *in vivo* genes, proteins and enzymes in the deep-sea still have a high ability. The discovery and research of the marine barophilic microorganisms provide a good foundation for further development on extreme enzymes[92,93].

There are some regions in oceans, where microorganisms from these locales are commonly highly acidophilic or alkalophilic: they can live in conditions of pH 5, even below pH 1, or alternatively over pH 9 conditions. Extracellular enzymes secreted by these microorganisms are commonly acidophilic enzymes (optimum pH < 3.0) or alkalophilic enzymes (optimum pH > 9.0). Compared with the neutral enzymes, the extreme pH enzymes show good stability in the environment, due to the particular enzyme molecule containinghigh proportion of acidic or basic amino acids. The enzymes, produced by acidophilic or alkalophilic microorganisms, could have wide applications for compound synthesis in extreme pH conditions [101–103]. In the ocean, the average seawater salinity is about 3.5%, but in some regions it could be higher. A large number of salt-tolerant or halophilic microorganisms will survive in these high salinity areas, and enzymes from halophilic microorganisms can maintain the stability at high salt concentrations [94,103].

## 5. Prospects

The 21st century is the century of the ocean, and the ocean is a vast treasure of human life. Recently, most countries face similar problems such as high population, resource consumption and pollution. Meanwhile, the marine biological progress and development gives a new source and options to humans. Marine microbial enzymes, especially marine extreme microbial enzymes, have become more and more important in applications.

Because enzymes have unequalled advantages, many industries are keenly interested in adapting enzymatic methods to the requirements of their processes. Clinical application of enzymes has been developing also. For example, surgeons used proteolytic enzymes for debridement of wounds, and promising clinical results have been reported by injection of certain enzymes such as streptokinase, crystallinetrypsin, and chymotrypsin. Since the increased therapeutic use of enzymes, presently unpredictable, rapid advances in this field may be expected[104].

Uses of enzymes have increased greatly during the past few years. Prospects are excellent for continuing to increase the usage of presently available enzymes in present applications, and in new uses, and also in the use of new enzymes for many purposes. also In addition, some new fields, like metagenomics, offer a powerful lens for viewing the microbial world that may have the potential to revolutionize our understanding of the marine microbial enzymes. Japan constantly increases its support to marine microbial enzymes research, and from 1992, the Japanese government made a series of marine microorganisms, planned to discover and clone proteins or enzymes with some special activity. In addition, Canada, Spain, Finland and Russia and other countries have also stepped up on marine bio-enzyme research.

Collectively, due to marine biological diversity and the specificity of biological metabolism, the study on a global scale is still just beginning, but it has huge potential for development and applications with industrial benefits.

## Figures and Tables

**Table 1:** Extremophiles and Living Environments.

Type	Living Environment	Genus
Psychrophilies	− 2~20 ºC	*Alteromonas, Algoriphagus, Psychrobacter*
Thermophilies	55~113 ºC	*Aquifex, Archaeoglobus, Bacillus, Hydrogenobacter, Methanococcus, Pyrococcus, Pyrodictium, Pyrolobus, Sulfolobus, Themococcus, Thermoproteus, Thermoplasma, Thermus, Thermotoga*
Acidophilies	pH < 4	*Acidianus, Desulfurolobus, Sulfolobus, Thiobacillus*
Alkaliphilies	pH > 9	*Natronobacterium, Natronococcus, Bacillus*
Halophilies	2~5 M NaCl	*Haloarcula, Halobacterium, Haloferax, Halorubrum*
